# Ss-Sl2, a Novel Cell Wall Protein with PAN Modules, Is Essential for Sclerotial Development and Cellular Integrity of *Sclerotinia sclerotiorum*

**DOI:** 10.1371/journal.pone.0034962

**Published:** 2012-04-25

**Authors:** Yang Yu, Daohong Jiang, Jiatao Xie, Jiasen Cheng, Guoqing Li, Xianhong Yi, Yanping Fu

**Affiliations:** 1 State Key Laboratory of Agricultural Microbiology, Huazhong Agricultural University, Wuhan, Hubei Province, People's Republic of China; 2 The Provincial Key Lab of Plant Pathology of Hubei Province, College of Plant Science and Technology, Huazhong Agricultural University, Wuhan, Hubei Province, People's Republic of China; Nanjing Agricultural University, China

## Abstract

The sclerotium is an important dormant body for many plant fungal pathogens. Here, we reported that a protein, named Ss-Sl2, is involved in sclerotial development of *Sclerotinia sclerotiorum*. Ss-Sl2 does not show significant homology with any protein of known function. Ss-Sl2 contains two putative PAN modules which were found in other proteins with diverse adhesion functions. Ss-Sl2 is a secreted protein, during the initial stage of sclerotial development, copious amounts of Ss-Sl2 are secreted and accumulated on the cell walls. The ability to maintain the cellular integrity of RNAi-mediated *Ss-Sl2* silenced strains was reduced, but the hyphal growth and virulence of *Ss-Sl2* silenced strains were not significantly different from the wild strain. *Ss-Sl2* silenced strains could form interwoven hyphal masses at the initial stage of sclerotial development, but the interwoven hyphae could not consolidate and melanize. Hyphae in these interwoven bodies were thin-walled, and arranged loosely. Co-immunoprecipitation and yeast two-hybrid experiments showed that glyceraldehyde-3-phosphate dehydrogenase (GAPDH), Woronin body major protein (Hex1) and elongation factor 1-alpha interact with Ss-Sl2. GAPDH-knockdown strains showed a similar phenotype in sclerotial development as *Ss-Sl2* silenced strains. Hex1-knockdown strains showed similar impairment in maintenance of hyphal integrity as *Ss-Sl2* silenced strains. The results suggested that Ss-Sl2 functions in both sclerotial development and cellular integrity of *S. sclerotiorum*.

## Introduction

*Sclerotinia sclerotiorum* (Lib.) de Bary is a notorious necrotrophic fungal pathogen that belongs to the Family *Sclerotiniaceae* in the Order *Helotiales*, and with worldwide distribution. This fungus may infect 450 plant species and subspecies in 75 families including many important crops, such as oilseed rape, sunflower, soybean, lettuce, celery and onion [1]. *Sclerotinia sclerotiorum* produces sclerotia, which are hard, asexual resting structure composed of compact vegetative hyphal cells. Sclerotia may survive for long periods under various adverse environmental conditions including low temperature, low moisture, UV irradiation and microbial activity [2]. Under the proper conditions, sclerotia may germinate myceliogenically to produce hyphae which infect host directly, often infect the stems or leaves near ground, or germinate carpogenically to produce apothecia. Apothecia may produce millions of airborne ascospores, which are the primary source of inocula in most Sclerotinia diseases [3], [4]. Therefore, sclerotial development is an important process in the lifecycle of *S. sclerotiorum*.

As an aggregate tissue, the sclerotium contains three distinct layers: a pigmented rind, a thin-walled cortex, and a large central medulla [5]. Sclerotial development has been divided into three distinguishable stages (1) initiation, (2) development, and (3) maturation [6], [7]. Based on the observation made under a variety of in vitro growth conditions, Li and Rollins redefined six distinct and sequential stages of sclerotial development: (S1) initiation, formation of discrete clusters of aerial hypha; (S2) condensation, sclerotial initials simultaneously condense and increase in size; (S3) enlargement, the sclerotial size increases at its highest rate; (S4) consolidation, sclerotial color is buff with a delimited surface; (S5) pigmentation, sclerotium is darkly pigmented; (S6) maturation, the sclerotium grows to full size and has a dark and hard surface [8].

Sclerotial development is a very complicated biological process. Many factors have been shown to play roles in modulating this process, such as the nutrient limitation, light, pH, temperature, mobilization of nutrients/metabolism, and oxidative stress [9]. Recently, the full genome sequence of *S. sclerotiorum* became available [10], and both PEG and *Agrobacterium*-mediated transformation systems for *S. sclerotiorum* have been established [11], [12], these progresses facilitate the study of *S. sclerotiorum* on molecular level. Some evidence for molecular mechanisms involved in sclerotial development has been described [13]–[18].

To further understand the mechanism of sclerotial development, gene expression profiles during hyphal and sclerotial growth were constructed using Illumina-Solexa sequencing (unpublished data). In this expression profile database, expression of genes involved in many biochemical processes showed significant differences. Many proteins with undefined functions also showed remarkable expression differences suggesting that these proteins also play important roles in sclerotial development. Characterization of these proteins may help us understand inherent mechanisms of sclerotial formation more profoundly.

The PAN module superfamily is widely found in proteins of bacteria, protozoa, oomycetes, plants, and animals, but not in fungi [19]. Modules in this family show low sequence identity and have been recognized largely by the conserved cystine pattern and observed or predicted secondary structure [20]. All members of this family contain a characteristic pattern of four cysteines (C1, C2, C3 and C4) that form two disulphide bridges: C1–C4, C2–C3. A subset of PAN modules (apple domain) appears in the plasma prekallikrein/coagulation factor XI family, and some proteins from *Caenorhabditis elegans* and plants possess an extra disulphide bridge that links the N and C termini. Many pieces of evidence indicated that proteins containing PAN modules show diverse adhesion functions, binding to protein or carbohydrate receptors [21].

A gene named *Ss-Sl2* (SSIG_05917, GenBank accession XM_001592945) showed high expression level during sclerotial development, with the relative expression during sclerotial development 400-fold greater than that at the hyphal growth stage. *Ss-Sl2* encodes a protein containing two domains which both showed structural similarity with PAN modules. In this study, *Ss-Sl2* was chosen for further analysis, and its biological role during sclerotial development was explored.

## Results

### The protein coded by *Ss-Sl2* has two putative PAN modules

**Figure 1:**
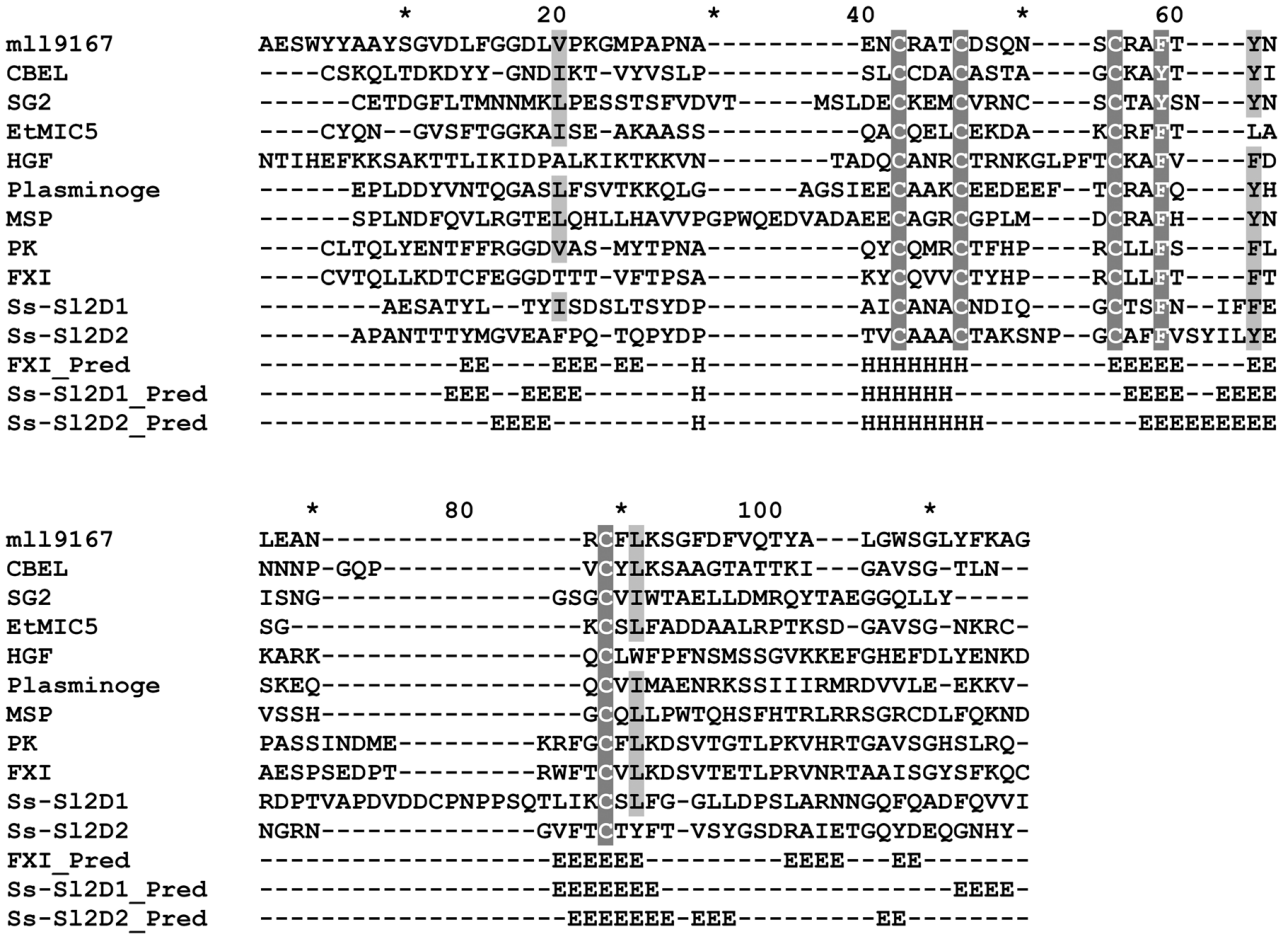
Multiple alignment of sequences of Ss-Sl2D1, Ss-Sl2D2 and representative PAN modules using ClustalX. The selected PAN modules are from a *Mesorhizobium loti* mll9167 protein (mll9167, BAB54559, residues 117–194), *Phytophthora parasitica* CBEL protein (CBEL, CAA65843, residues 197–268), *Ipomoea trifida* secreted glycoprotein 2 (SG2, AAA97902, residues 348–425), *Eimeria tenella* microneme protein 5 (EtMIC5, CAB52368, residues 713–781), human macrophage stimulating protein (MSP, BAH12774, residues 21–105), human plasminogen (Plasminogen, AAA36451, residues 20–98), human hepatocyte growth factor (HGF, AAA52648, residues 37–123), human prekallikrein (PK, AAY40900, residues 21–103), and human coagulation factor XI (FXI, AAA51985, residues 20–103). Conserved amino acids are shown with a shaded background. The secondary structural elements (H for alpha helix, E for beta strand) from the PAN/apple domains of FXI (FXI_Pred), Ss-Sl2D1 (Ss-Sl2D1_Pred) and Ss-Sl2D2 (Ss-Sl2D1_Pred) predicted with the PSIPRED program are the last three sequences in the multiple alignments.

*Ss-Sl2* is predicted to encode a protein which contains 352 amino acids, the molecular weight is near 34.14 kDa and the isoelectric point is close to 4.72. Ss-Sl2 has an N-terminal signal peptide and cleavage site is between amino acid 16 and 17 predicted with SignalP 3.0 [22]. Using a cut-off expect value of 10^−5^, homologs of Ss-Sl2 can be found in some ascomycetes species and a basidiomycete, *Ustilago maydis*, and all of these homologs are of unknown function. No conserved protein domains were found in Ss-Sl2 with biotools including Pfam, Smart, ProSite Scan, whereas a close examination of the sequences of Ss-Sl2 revealed the structure of two domains, namely Ss-Sl2D1 (amino acid 138–225) and Ss-Sl2D2 (amino acid 256–336) are similar to that of PAN domain. The sequences of Ss-Sl2D1 and Ss-Sl2D2 were used in a search of hidden Markov model (HMM) profiles for potential structural homologs in the Protein Data Bank, using the HHpred structure-prediction server [23], [24]. For Ss-Sl2D1, the top-ranked hits is PAN module in hepatocyte growth factor (HGF), with an E-value of 0.76 (P-value of 3E-05) and a probability score of 79.2. The second-ranked hits is PAN module in coagulation factor XI (FXI), with an E-value of 2.4 (P-value of 9.4E-05) and a probability score of 73.3. For Ss-Sl2D2, the top-ranked hits is PAN module in coagulation factor XI (FXI), with a modest E-value of 3.7 (P-value of 0.00014) and a probability score of 63.7. As shown in the bottom line of Figure 1, the positions and types of the predicted secondary structure elements of these two domains are in good agreement with the typical PAN domain in FXI. Based on the structural prediction, the alignment of the sequences of typical PAN modules was constructed. As shown in Figure 1, the four cysteines conserved in Ss-Sl2D1 and Ss-Sl2D2 align with the four cysteines conserved in all PAN modules. Collectively, the above observations suggested that the Ss-Sl2D1 and Ss-Sl2D2 are homologous with the PAN module.

### Ss-Sl2 in cell walls of *S. sclerotiorum*

**Figure 2:**
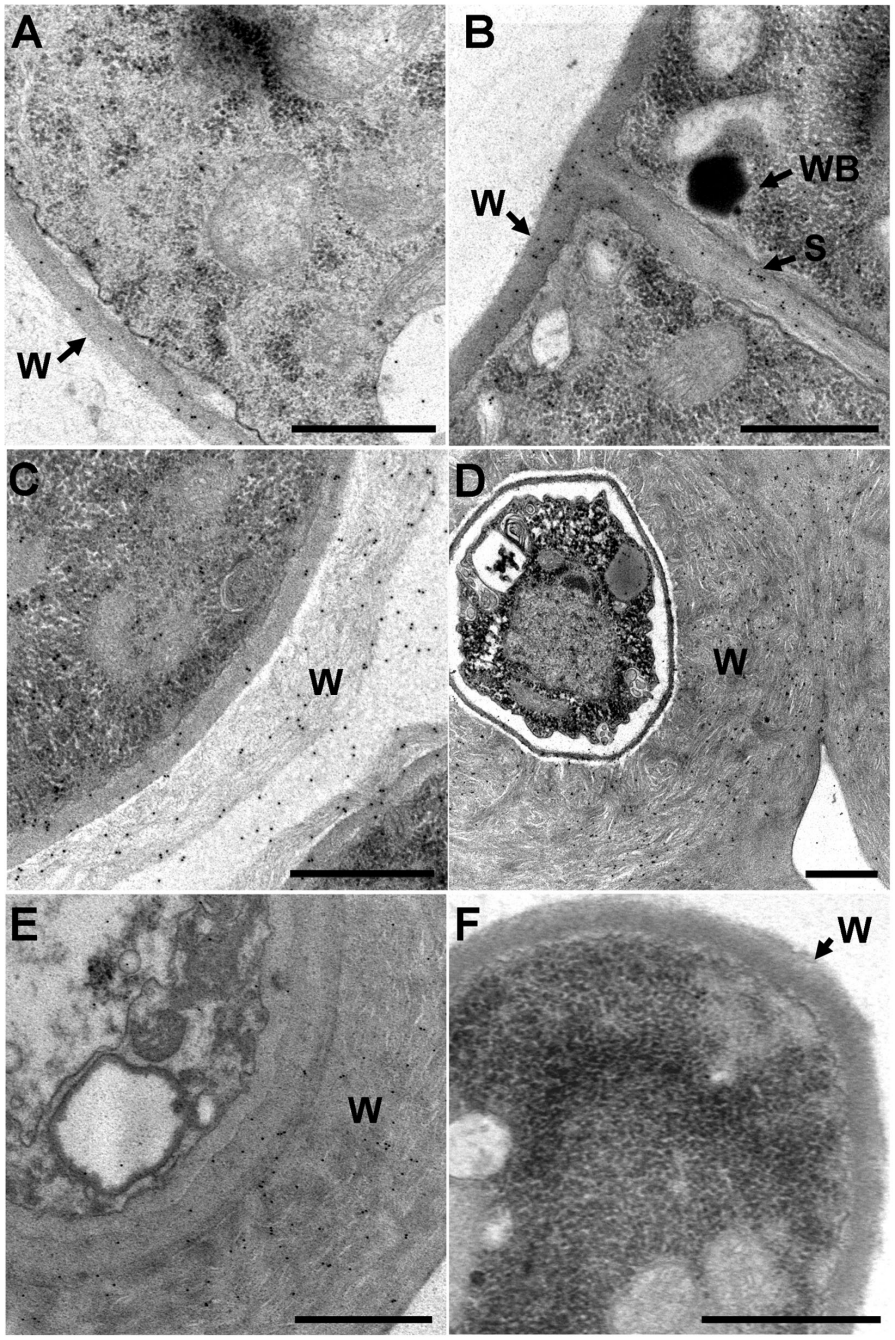
Subcellular location of Ss-Sl2 in *S. sclerotiorum*. Immunogold labeling of ultrathin sections from (A) hyphae at the early stage of vegetative growth (cultured on PDA plates for 1 day), (B) hyphae at the later stage of vegetative growth (cultured for 3 days), (C) sclerotial initial (cultured for 4 days), (D) sclerotia just starting to accumulate melanin and consolidate (cultured for 6 days), and (E) mature sclerotia (cultured for 7 days) with anti-Ss-Sl2 polyclonal antibodies, or from hyphae with preimmune serum (F) are shown. The gold particles are visible on the cell walls and septa in a patchy distribution. W, cell wall; S, septa; WB, Woronin body. Bar = 1 μm.

**Figure 3:**
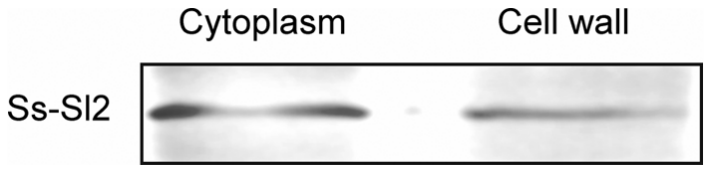
Ss-Sl2 was detected in cytoplasm and cell wall of *S. sclerotiorum*. The cell wall protein and cytoplasm protein in mycelium were extracted respectively (50 μg) and subjected to western blot analysis with anti-Ss-Sl2 polyclonal antibodies.

Protein structure prediction showed Ss-Sl2 has a typical signal peptide at the N-terminal, indicating that Ss-Sl2 acts possibly as a secretory protein. This prediction was further confirmed by immunoelectron microscopy with anti-Ss-Sl2 polyclonal antibodies (Figure 2). Results revealed that at the early vegetative growth stage, only a few of gold particles could be observed dispersed in the cell walls of young hyphae. At the later stage of hyphal growth, more gold particles can be found in the cell walls. There were also some gold particles located at the septa of hyphae. At the initial stage of sclerotial development, hyphae tended to aggregate and form discrete clusters of aerial hyphae. In this case, the hyphal cell wall was surrounded by an electron-transparent fibrous layer, and large numbers of gold particles were observed. In the consolidation and maturation stages, the fibrous layer became compact, melanin was deposited and Ss-Sl2 was widely distributed on this electron-dense cell surface. The control section treated with preimmune serum displayed no labeling over any parts of the cell. The subcellular location of Ss-Sl2 was also further confirmed by western blot analysis. As shown in Figure 3, Ss-Sl2 could be detected in the cell wall parts of hyphae in *S. sclerotiorum*.

### High expression of *Ss-Sl2* during sclerotial development

**Figure 4:**
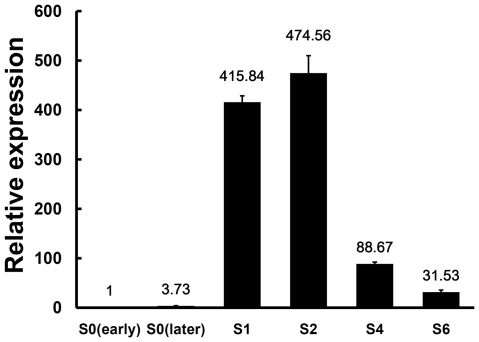
Real-time RT-PCR analysis of Ss-Sl2 transcript in different sclerotial developmental stages of *S. sclerotiorum*. S0 (early)  =  the early stage of vegetative growth (cultured on PDA plates for 1 day); S0 (later)  =  the later stage of vegetative growth (cultured for 3 days); S1 =  the initiation stage of sclerotial development (cultured for 4 days); S2 =  condensation stage (cultured for 5 days); S4 =  consolidation stage (cultured for 6 days); S6 =  maturation stage (cultured for 7 days). The expression level of *Ss-Sl2* cDNA measured by RT-PCR was normalized to that of *actin* cDNA in extracts from each developmental stage. The abundance of cDNA from S0 (early) samples was assigned a value of 1. Bars indicate standard error.

The expression levels of *Ss-Sl2* during different morphological stages of sclerotial development of *S. sclerotiorum* were determined with a real-time reverse-transcriptase (RT)-PCR approach. At the initial stage of sclerotial development, the level of *Ss-Sl2* expression had a dramatic increase and it reached the highest level at the condensation stage, which was approximately 470-fold greater than during early stage of vegetative hyphal growth (Figure 4). As the sclerotia matured gradually, the extent of *Ss-Sl2* expression gradually declined, but remained higher than during hyphal growth. This result suggests that Ss-Sl2 is involved in or related to sclerotial development in *S. sclerotiorum*.

### RNA interference-mediated down-regulation of *Ss-Sl2* impairs sclerotial development

**Figure 5:**
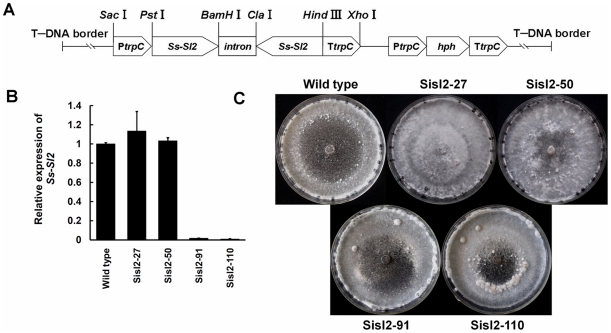
Construction of an Ss-Sl2 RNAi vector (pSisl2) and functional analysis of *Ss-Sl2* silenced strains. (A) A 310 bp fragment of *Ss-Sl2* was inserted in the sense orientation between an *Aspergillus nidulans trpC* promoter and an intron from *Gibberella zeae*, and the same fragment of *Ss-Sl2* was inserted in antisense orientations between this intron and the *trpC* terminator. (B) Expression level of *Ss-Sl2* in isolates containing pSisl2 and in the wild type strain were determined by real-time RT-PCR. The expression level of *Ss-Sl2* cDNA was normalized to that of *actin* cDNA in extracts from each strain. The abundance of cDNA from the wild type was assigned a value of 1. Bars indicate standard error. (C) Phenotype of the wild type strain and *Ss-Sl2* gene-silenced transformants.

To confirm whether Ss-Sl2 is a key factor for sclerotial development of *S. sclerotiorum*, this gene was silenced with the RNAi technique. A gene silencing vector, pSisl2, was constructed (Figure 5A) and used to transform the wild type mycelia of *S. sclerotiorum*. Thirty-five independent transformants were obtained and confirmed through the amplification of the hygromycin resistance gene *hph*. Real-time RT-PCR was used to assess the abundance of *Ss-Sl2* transcripts in the different transformants and the result showed that Sisl2-91 and Sisl2-110 exhibited markedly reduced expression levels of *Ss-Sl*2 (Figure 5B). Thus, Sisl2-91 and Sisl2-110 were used for further research.

**Figure 6:**
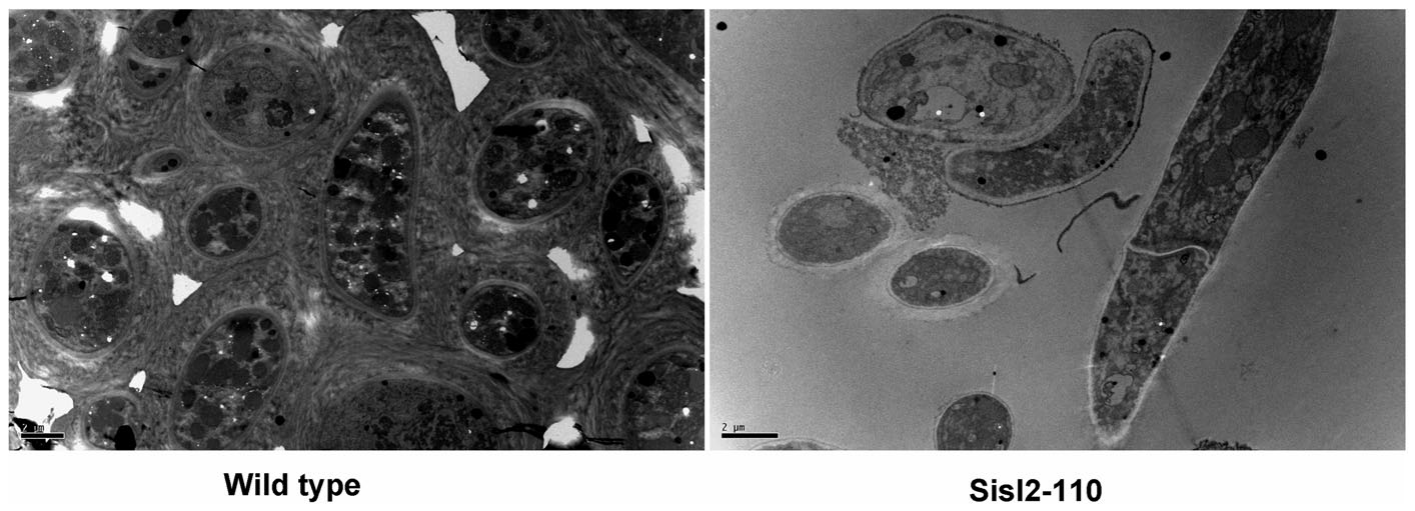
Ultrastructural analysis of sclerotia produced by the wild type and Sisl2-110. The inner structure of sclerotia produced by the wild type showing a compact structure and by Sisl2-110 showing a loose structure, both observed by TEM. Bar = 2 μm.

Sisl2-91 and Sisl2-110 have similar growth rates on PDA plates and similar levels of virulence on detached rapeseed leaves or on living plants of *Arabidopsis thaliana* as the wild type strain. Unlike the wild type strain, these two transformants could not produce typical sclerotia on PDA, and just formed distinct clusters of aerial hyphae at the edge of the plates. Furthermore, these hyphal masses did not develop into mature sclerotia (Figure 5C). Cells in these abnormal sclerotia were thin-walled, and loosely arranged, while cell walls were thick and cells tightly compressed in sclerotia of the wild type strain (Figure 6). In addition, the cells in mature sclerotia were surrounded by thicken cell walls which consisted of an inner original hyphal wall and an outer compact fibrous structure of very high electron density, while the fibrous layer surrounding the abnormal sclerotial cells were still electron-transparent and melanin-deficient. Thus, *Ss-Sl2* is essential for sclerotial development, and silencing the expression of *Ss-Sl2* prevented sclerotia from maturing.

### Ss-Sl2 is involved in melanin biosynthesis of *S. sclerotiorum*

**Figure 7:**
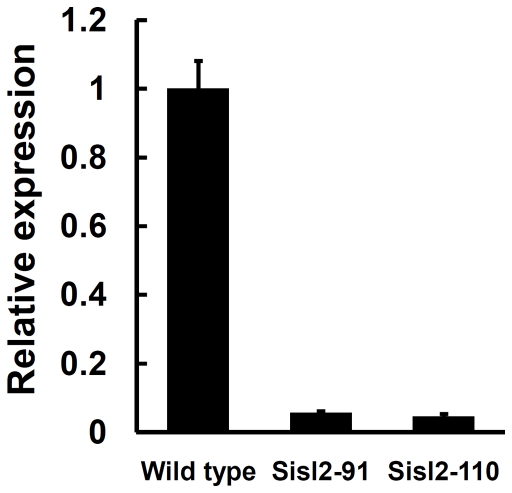
Expression level of a melanin biosynthesis associated polyketide synthase-encoding gene (*Ss-Pks1*) in *Ss-Sl2* gene silenced strains of *S. sclerotiorum* by real-time RT-PCR. The expression level of *Ss-Pks1* cDNA was normalized to that of *actin* cDNA in extracts from each strain. The abundance of cDNA from the wild type was assigned a value of 1. Bars indicate standard error.

The *Ss-Sl2* silenced strains could produce hyphal masses, but the white hyphal masses could not pigment to black color, Ss-Sl2 is likely to be involved in the production of melanin during sclerotial development. We identified a melanin biosynthesis associated polyketide synthase-encoding gene (*Ss-Pks1*) in *S. sclerotiorum*, and Real-time RT-PCR analysis results indicated that the expression level of *Ss-Pks1* in *Ss-Sl2* silenced strains was significant lower than that in the wild type strain (Figure 7). Thus, Ss-Sl2 has a function on the biosynthesis of melanin in *S. sclerotiorum* through regulating the expression of *Ss-Pks1*.

### Ss-Sl2 is involved in the maintenance of cell integrity in hyphae

**Figure 8:**
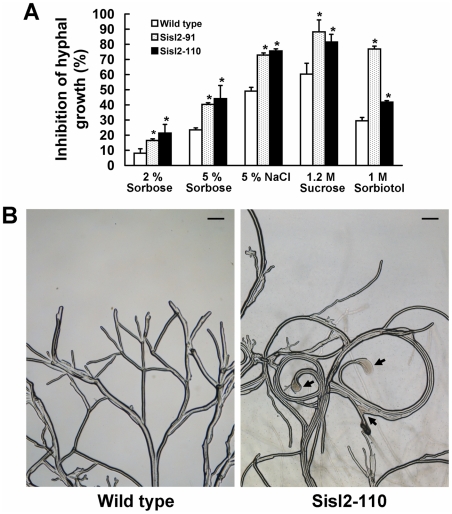
Growth characteristics and analysis of *Ss-Sl2* silenced strains of *S. sclerotiorum*. (A) The inhibition of hyperosmotic stress and sorbose to the hyphal growth rate of *Ss-Sl2* silenced strains and the wild type. *Significantly different from the wild type strain (*P*<0.05). (B) Cytoplasmic bleeding at the hyphal tips of Sisl2-110 when cultured on medium with 5% sorbose for 2 d. Bar = 50 μm.

The ability of maintaining the cellular integrity in hyphae was compared between the *Ss-Sl2* silenced strains and the wild type. Under hyperosmotic stress, the hyphal growth of *Ss-Sl2* silenced strains was more greatly suppressed than that the wild type. When growing on PDA plates amended with 5% NaCl, 1.2 M sucrose or 1 M sorbitol, the inhibition of hyphal growth was significantly greater for Sisl2-110 and Sisl2-91 than the wild type (Figure 8A). The ability of maintaining the cellular integrity was further evaluated by comparing the effects of sorbose on hyphal growth rate. Comparative growth assays showed that Sisl2-110 and Sisl2-91 were more sensitive to sorbose than the wild type (Figure 8A). Moreover, cytoplasmic bleeding at mycelium tips was observed frequently in Sisl2-110 on 5% sorbose, while such the phenomenon only appeared occasionally in the wild type (Figure 8B). The results indicated that Ss-Sl2 contributes to maintain the cell integrity in hyphae.

### Ss-Sl2 interacts with glyceraldehyde-3-phosphate dehydrogenase and Woronin body major protein

**Figure 9:**
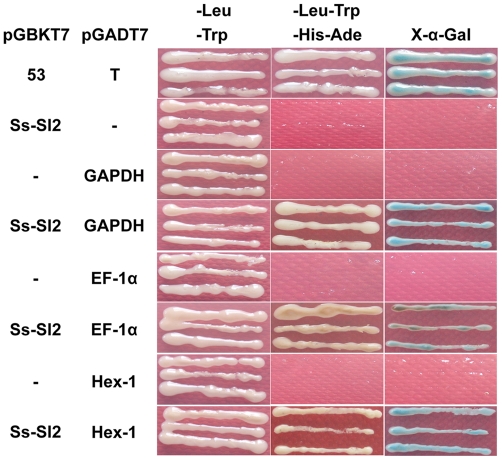
Interaction of Ss-Sl2 and GAPDH, EF-1α and Hex1 in yeast Y2HGold cells. The *Ss-Sl2* region code for amino acid 17–352 (without signal peptide) was inserted into pGBKT7 to get the bait plasmid. The cDNA of GAPDH, EF-1α and Hex1 were inserted into pGADT7 to obtain the prey plasmids. The prey plasmids respectively were co-transformed into Y2HGold with the bait plasmid. Transformed Y2HGold cells grown on the SD/-Leu-Trp, SD/-Leu-Trp-His-Ade and SD/-Leu-Trp-His-Ade with X-α-gal. pGBKT7 and pGADT7 are bait and prey vectors without inserts. pGBKT7-53 and pGADT7-T encode two fusion proteins that are known to interact (Clontech).

To further understand the mechanism of Ss-Sl2 affecting sclerotial development in *S. sclerotiorum*, co-immunoprecipitation (CO-IP) with Ss-Sl2 antibodies were used to investigate that interacted with Ss-Sl2 on a proteome scale. Several protein binds were detected in the sample buffer with Ss-Sl2 antibodies but were absent in that with pre-immune serum. These bands were analyzed by LC/MS/MS and 19 proteins appeared twice in two independent experiments (Table 1). Nine of these are ribosomal proteins, which are assumed to play an essential role in the protein synthesis. The remaining proteins, including glyceraldehyde-3-phosphate dehydrogenase (GAPDH), elongation factor 1-alpha (EF-1α), Woronin body major protein (Hex1), heat shock protein 90 (HSP90), heat shock protein 60 (HSP60), heat shock protein 70 (HSP70), ATP synthase beta chain (ATPB), histone H4.1 (H4), histone H2B.1 (H2B) and actin, were chosen to investigate their direct interaction with Ss-Sl2 with the yeast two-hybrid system. The results showed that only three proteins, namely GAPDH, Hex1 and EF-1α could interact with the Ss-Sl2 directly, and EF-1α was found to have only a weak interaction with Ss-Sl2 (Figure 9). The expression level of the GAPDH-encoding gene *Ss-Gpd* (SS1G_07798, GenBank accession XM_001591123) and the Hex1-encoding gene *Ss-Hex1* (SS1G_03527, GenBank accession XM_001595388) were detected in *Ss-Sl2* silenced strains with real-time RT-PCR. The expression level of *Ss-Gpd* and *Ss-Hex1* in Sisl2-91 and Sisl2-110 showed no difference from that in the wild type.

**Table 1:** Summary of Ss-Sl2 interacting proteins identified by Ss-Sl2 antibodies co-immunoprecipitation and LC/MS/MS analysis.

Accession number	Protein description (# of peptides)^a^	Percent coverage^b^	MW (Da)
XP_001592594	60S ribosomal protein L35 (6)	60.6%	14513
XP_001585386	Histone H4.1 (5)	59.2%	11363
XP_001587720	Histone H2B.1 (7)	59.0%	14774
XP_001595438	Woronin body major protein (6)	56.0%	18262
XP_001585908	40S ribosomal protein S2 (8)	51.9%	28410
XP_001591676	Ribosomal protein S3 (8)	40.3%	28652
XP_001586490	40S ribosomal protein S7 (4)	34.3%	22444
XP_001591173	Glyceraldehyde-3-phosphate dehydrogenase (9)	31.1%	36864
XP_001586516	60S ribosomal protein L17 (5)	30.5%	20938
XP_001594074	60S ribosomal protein L10-A (5)	29.4%	25641
XP_001596964	60S ribosomal protein L9 (4)	27.7%	21738
XP_001589969	Actin (6)	24.5%	41841
XP_001597891	Heat shock protein 60 (9)	23.1%	60934
XP_001594091	Elongation factor 1-alpha (8)	20.9%	50304
XP_001598862	40S ribosomal protein S24 (2)	20%	15458
XP_001596961	60S ribosomal protein L6 (3)	19.5%	22125
XP_001591168	ATP synthase beta chain (5)	17.4%	55642
XP_001598048	Heat shock protein 70 (6)	14.6%	68661
XP_001591945	Heat shock protein 90 (7)	13.9%	79529

a # of peptides refers to the combined number of peptides identified for the protein in two samples.

b Percent coverage refers to percent of MS/MS peptide coverage of identified protein seen over the entire amino-acid sequence.

### Knockdown of *Ss-Gpd* impairs sclerotial formation

**Figure 10:**
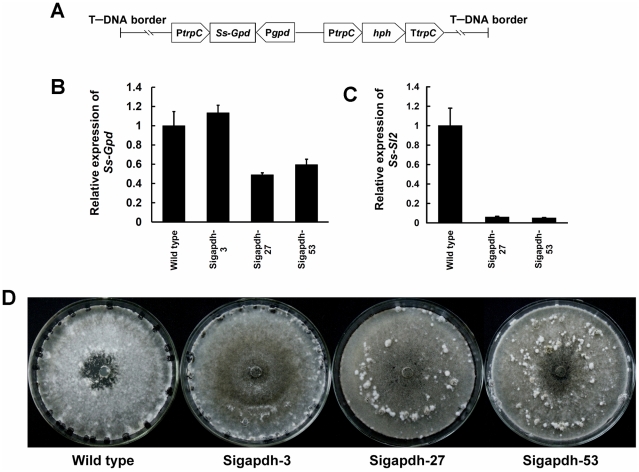
Construction of *Ss-Gpd* RNAi vector (pSigapdh) and analysis of *Ss-Gpd* silenced strains. (A) A 538-bp fragment of *Ss-Gpd* was inserted between the *N. crassa trpC* promoter P*trpC* and *A. nidulans gpd* promoter P*gpd*. (B) Expression level of *Ss-Gpd* in isolates containing the pSigapdh and in the wild type determined by real-time RT-PCR. The expression level of *Ss-Gpd* cDNA was normalized to that of *actin* cDNA in extracts from each strain. The abundance of cDNA from the wild type was assigned a value of 1. Bars indicate standard error. (C) Expression level of *Ss-Sl2* in *Ss-Gpd* silenced strains and in the wild type determined by real-time RT-PCR. The expression level of *Ss-Sl2* cDNA was normalized to that of *actin* cDNA in extracts from each strain. The abundance of cDNA from the wild type was assigned a value of 1. Bars indicate standard error. (D) Phenotype of the wild type and *Ss-Gpd* gene-silenced transformants.

The gene *Ss-Gpd* coding for GAPDH of *S. sclerotiorum* was specifically knocked down using the RNAi technique. *Ss-Gpd* RNAi vector, pSigpd, was constructed (Figure 10A) and used to transform the wild type strain of *S. sclerotiorum*. Twenty transformants were isolated and verified by PCR amplification. Among these transformants, the abundance of *Ss-Gpd* transcripts of Sigapdh-27 and Sigapdh-53 was reduced compared with that of the wild type (Figure 10B). Because GAPDH interacted with Ss-Sl2 directly, expression of *Ss-Sl2* in *Ss-Gpd* silenced strains was detected. The expression level of *Ss-Sl2* in Sigapdh-27 and Sigapdh-53 was much lower than that of the wild type (Figure 10C). When Sigapdh-27 and Sigapdh-53 were cultured on PDA plates, they did not produce typical sclerotia, but only formed interwoven hyphal masses (Figure 10D). The phenotype of *Ss-Gpd* silenced transformants is very similar to that of *Ss-Sl2* silenced transformants, with respect to sclerotial development.

### Hex1 functions in the maintenance of cellular integrity

**Figure 11:**
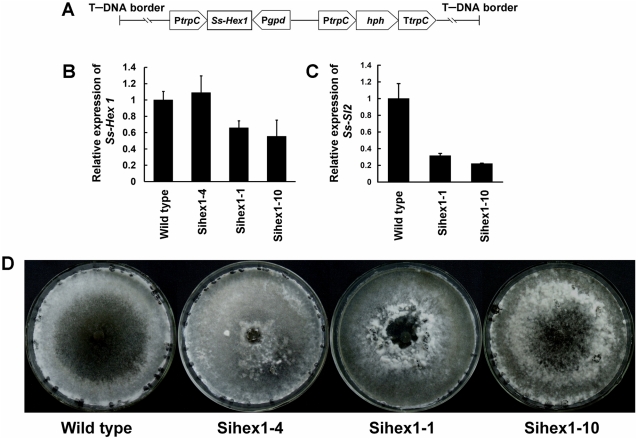
Functional analysis of *Ss-Hex1* with RNAi technology. (A) A 465-bp fragment of *Ss-Hex1* was inserted between the *N. crassa trpC* promoter P*trpC* and *A. nidulans gpd* promoter P*gpd*. (B) Expression level of *Ss-Hex1* in strains containing the pSihex1 and wild type strain using the real-time RT-PCR. The expression level of *Ss-Hex1* cDNA was normalized to that of *actin* cDNA in extracts from each isolate. The abundance of cDNA from the wild type was assigned a value of 1. Bars indicate standard error. (C) Expression level of *Ss-Sl2* in *Ss-Hex1* silenced strains and in the wild type determined by real-time RT-PCR. The expression level of *Ss-Sl2* cDNA was normalized to that of *actin* cDNA in extracts from each strain. The abundance of cDNA from the wild type was assigned a value of 1. Bars indicate standard error. (D) Phenotype of the wild type and *Ss-Hex1* gene-silenced transformants.

**Figure 12:**
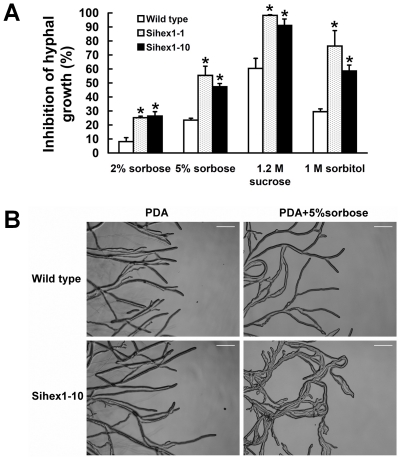
Growth characteristics and analysis of *Ss-Hex1* silenced strains. (A) The inhibition of hyperosmotic stress and sorbose to the hyphal growth rate of *Ss-Hex1* silenced strains and the wild type. *Significantly different from the wild type strain (*P*<0.05). (B) Microscopic observation of hyphal tips and forming branches for Sihex1-10 and the wild type on PDA and PDA with 5% sorbose. Bar = 100 μm.

Hex1 is a major structural protein of Woronin bodies which functions in the maintenance of cellular integrity in response to cellular damage [25]–[30]. Silenced strains of *Ss-Hex1* were obtained using the RNAi technique. Among the 15 independent transformants, the expression of *Ss-Hex1* in Sihex1-1 and Sihex1-10 were reduced compared with that in the wild type (Figure 11B). The expression level of *Ss-Sl2* in *Ss-Hex1* silenced strains were also detected and the result showed that the transcript abundance of *Ss-Sl2* in Sihex1-1 and Sihex1-10 were less as compared to that in the wild type (Figure 11C). The mycelial growth of Sihex1-1 and Sihex1-10 showed no apparent differences with that of the wild type on PDA. The repressed expression of *Ss-Hex1* partially influenced sclerotial development, since Sihex1-1 and Sihex1-10 produced fewer sclerotia than the wild type, and these sclerotia showed a non-regular distribution on PDA plates (Figure 11D). *Ss-Hex1* silenced strains have an impaired in maintenance of cellular integrity. As shown in Figure 12A, the inhibition of hyphal growth was significantly greater for Sihex1-1 and Sihex1-10 than the wild type on medium with sorbose, and also under the hyperosmotic stress. Microscopic observation showed that the phenotype of hyphal tips and forming branches for Sihex1-10 was more abnormal than that for the wild type on medium with 5% sorbose (Figure 12B). The phenotype of *Ss-Hex1* silenced transformants for cell integrity was similar to that of *Ss-Sl2* silenced transformants.

## Discussion

In this research we identified a gene, named *Ss-Sl2*, which encodes a secretory protein that shows no homology with any known functional protein from *S. sclerotiorum*. We demonstrated that Ss-Sl2 can play an important role in sclerotial development and the maintenance of hyphal cell integrity. The targeted knockdown of gene expression of *Ss-Sl2* resulted in an early premature abortion of sclerotial development and impairment of hyphal cell integrity. Ss-Sl2 is not likely involved in the pathogenicity of *S. sclerotiorum* since RNAi-silenced transformants had similar virulence levels compared to the wild type strain.

Secondary structure analysis of Ss-Sl2 revealed the existence of two putative PAN modules which are composed of four cysteine residues at highly conserved positions. On the basis of homology we may thus predict that disulphide bond patterns in Ss-Sl2D1 and Ss-Sl2D2 are similar to that in other PAN modules. PAN modules (including apple domains) have been described previously in glycoproteins such as coagulation factor XI [31], hepatocyte growth factor [21], *Phytophthora parasitica* lectin protein CBEL [19], [32], *Toxoplasma gondii* micronemal protein MIC4 [33], or *Eimeria tenella* mironeme protein EtMIC5 [34]. Several studies have shown that PAN modules have carbohydrate-binding activities [35]. The PAN module in CBEL has cellulose-binding activities and closely resembles the fungal cellulose binding domain (CBD) [19], [32]. Interestingly, Ss-Sl2 and CBEL share common features, notably their cell wall localization, and the presence of two PAN modules. It is not known whether the PAN module in Ss-Sl2 might bind carbohydrates. The phenomenon that a large amount of Ss-Sl2 located at the thickened cell walls which consist of fiber at the initial stage of sclerotial development indicated that PAN modules in Ss-Sl2 may share similar activity with that in CBEL.

**Table 2:** Primers used in plasmids construction in yeast two-hybrid system.

Primers	Primers Sequences	Restriction enzyme	Constructs
BD-sl2F	5′-CGCCATATGAATCCGTTCCTCAAACCT-3′	*Nde*I	pGBKT7-Sl2
BD-sl2R	5′-CGGTCGACGGTATGATGTGTAACCAT-3′	*Sal*I	pGBKT7-Sl2
GAPDH-ADF	5′-GCGGATCCATGGCTCCTACTAAAGTTGG -3′	*BamH*I	pGADT-GAPDH
GAPDH-ADR	5′-CCGCTCGAGCTGCTTGTTATCAACCTTG -3′	*Xho*I	pGADT-GAPDH
EF1α-ADF	5′-CGGAATTCATGGGAAAGGAAAAGACACA-3′	*EcoR*I	pGADT-EF-1α
EF1α-ADR	5′-GCGGATCCTTTCTTGGCACCAGCCTT-3′	*BamH*I	pGADT-EF-1α
Hex1-ADF	5′-CCGGAATTCATGGGTTACTACGATGAAGA -3′	*EcoR*I	pGADT-Hex1
Hex1-ADR	5′-CCATCGATTAGACGGGATCCGTGGAC -3′	*Cla*I	pGADT-Hex1
HSP60-ADF	5′-CGGAATTCATGCAACGAGCTTTGAGTTCTA-3′	*EcoR*I	pGADT-HSP60
HSP60-ADR	5′-CCATCGATACCCATTCCTCCCATTCCTC-3′	*Cla*I	pGADT-HSP60
HSP70-ADF	5′-CCGGAATTCATGGCTCCAGCTATTGGTAT -3′	*EcoR*I	pGADT-HSP70
HSP70-ADR	5′- GCGGATCCGTCAACCTCTTCGATCTCA-3′	*BamH*I	pGADT-HSP70
HSP90-ADF	5′- CCATCGATATGGCTGGCGAAACTTTTG -3′	*Cla*I	pGADT-HSP90
HSP90-ADR	5′- CGGGATCCGTCAACCTCCTCCATAGC -3′	*BamH*I	pGADT-HSP90
ATPB- ADF	5′- CGGAATTCATGTTCAGGAGCGCCATC -3′	*EcoR*I	pGADT- ATPB
ATPB- ADR	5′- GCTCTAGAGCCCCTTCCCTTTTTCATCATC -3′	*Xba*I	pGADT- ATPB
Actin-ADF	5′- CGGAATTCATGGAAGAAGAAGTCGCAG -3′	*EcoR*I	pGADT-Actin
Actin-ADR	5′- GCTCTAGAGGAAACACTTGCGGTGGACA-3′	*Xba*I	pGADT-Actin
H2B-ADR	5′- CGGAATTCATGCCACCAAAGGGAGTT -3′	*Cla*I	pGADT-H2B
H2B-ADF	5′-CCATCGATTTTCGTGGATGAAGAGTACTTG -3′	*BamH*I	pGADT-H2B
H4-ADR	5′- CGGAATTCATGACTGGACGCGGAAAG -3′	*EcoR*I	pGADT-H4
H4-ADF	5′- CCATCGATACCACCGAAACCATAGAGGG -3′	*Cla*I	pGADT-H4

Many proteins that contain PAN modules putatively function in the adherence process, such as CBEL [32], MIC4 [33], or EtMIC5 [34]. Strains of *P. parasitica* with suppressed *CBEL* expression showed severely impaired adhesion to a cellophane membrane, differentiation of lobed structures in contact with cellophane, and formation of branched aggregating hyphae on cellophane and on flax cellulose fibers [36]. Information on proteins containing PAN modules in fungi is scarce. *Ss-Sl2-*knockdown causes termination of sclerotial development at the condensation stage which normally involves adhesive substances. During the initiation of sclerotial development, hyphae of wild type *S. sclerotiorum* adhere and form a more condensed sclerotial initial body; this process may need a mucilage-like substance which may function as a hyphal adhesive [37]. However, proteins that work as mucilage-like substances in sclerotial development have not been identified. Our observation showed that a large amount of Ss-Sl2 was secreted and located at the cell surface during initial stages of sclerotial development. In *Ss-Sl2*-knockdown transformants, the hyphae in proto-sclerotial bodies were arranged loosely, the outer cell wall layer was very thin, and hyphae were not in tight contact with each other. Although proteins with PAN modules may work as adhesives, the function of Ss-Sl2 as a hyphal adhesive needs further confirmation.

The accumulation of melanin in sclerotia is very important for *S. sclerotiorum* to survive in soil; the melanized out layer of sclerotia has a protective function against both biotic and abiotic stress [2]. Melanization is a marker for the sclerotial maturation; usually mature sclerotia show dense black color. Our finding suggested that Ss-Sl2 has a function not only on hyphal masses consolidation, but also on pigmentation indirectly, two key processes for sclerotial development.

Hyphal adhesion and condensation during sclerotial development of *S. sclerotiorum* are likely to be very complicated processes. Co-immunoprecipitation experiment results showed that many proteins interacted with Ss-Sl2 directly or indirectly. Among them, two proteins, namely GAPDH and Hex1 were shown to interact strongly with Ss-Sl2 through yeast two-hybrid assay, and a protein, namely EF-1α, interacted weakly. The typical function of GAPDH in organisms is for glycolysis in the cytoplasm; however, GAPDH has been reported to be distributed on the cell wall and function in cell adhesion to host, as has been reported for *Candida albicans* [38], *Escherichia coli* [39], and *Paracoccidioides brasiliensis* [40]. In *Kluyveromyces marxianus*, the cell wall-associated GAPDH was involved in the adhesion between cells leading to flocculation [41], [42]. Like GAPDH, EF-1α has also been found on the cell surface and to play an important role in adhesive processes in many species, including tobacco [43], *Lactobacillus johnsonii* [44], *Mycoplasma pneumoniae* [45]. Targeted knockdown of the expression of *Ss-Gpd* resulted in abortion of sclerotial development, since transformants formed only loose interwoven hyphal masses, which displayed high similarity with the *Ss-Sl2* silenced strains. Thus, we suggest that sclerotial development may require cooperation between Ss-Sl2 and cell surface GAPDH, which work together and contribute to the cell-cell adhesive process at the initial stage of sclerotial development.

Though Ss-Sl2 showed a low level of accumulation at the stage of hyphal growth, it is likely to have a function in the maintenance of hyphal cell integrity, because *Ss-Sl2* silenced transformants were more sensitive to the sorbose and showed frequent cytoplasmic bleeding at mycelial tips when cultured on medium with sorbose. We found that Ss-Sl2 interacts with Hex1 directly. Hex1 is a major structural protein of Woronin bodies and functions in the maintenance of cellular integrity [26], [27], [29]. In *M. oryzae*, the *HEX1* deletion strain showed poor hyphal growth, and hyphal tips displayed frequent cytoplasmic bleeding when cultured on medium containing 2% sorbose [29]. Our study showed that *Ss-Hex*1 silenced strains were more sensitive to sorbose, indicating that the function of Hex1 in *S. sclerotiorum* is similar to that in other filamentous ascomycetes. *Ss-Hex1* silenced strains showed poor and restricted growth on the medium with 5% sorbose, but no frequent cytoplasmic leaks were observed as that in *N. crassa* [27] and *M. grisea* [29]. One possible reason is that Sihex1-1 and Sihex1-10 are gene-silenced strains in which the expression of *Ss-Hex1* is just partly suppressed. The direct interaction between the Ss-Sl2 and Hex1 leads us to speculate that the capacity of Ss-Sl2 to maintain the integrity of cell may be related to Hex1.

In wounded hyphae, cytoplasmic flow causes Woronin bodies to occupy septal pore, and subsequently prevent the loss of cytoplasm [46]. After occlusion of the septal pore, a wounded membrane is resealed by the deposition of cell wall over the Woronin body septal-pore complex and regeneration of a new hyphal tip from the occluded septum [25]. So far, the mechanism of membrane resealing at the septal pore achieved is still unknown [30]. The identification of Woronin body-associated proteins may provide insight into events that immediately follow occlusion of the septal pore [26]. In this research, Ss-Sl2 was located near septa and has a direct interaction with Hex1. Ss-Sl2 contains two putative PAN modules which may mediate protein-carbohydrate interactions. We speculate that Ss-Sl2 is likely to be a protein involved in the anchoring of Woronin bodies on fungal membranes, but more evidence is needed.

In conclusion, we characterized a fungal protein with PAN modules, and we found that this protein plays an important role in sclerotial development and hyphal cell integrity of *S. sclerotiorum*. Ss-Sl2 may cooperate with GAPDH and other proteins to adhere and condense hyphae of *S. sclerotiorum* in the sclerotial maturation process. It may also anchor Woronin bodies through binding with Hex1 to maintain cell integrity. Most research demonstrates that sclerotial development in *S. sclerotiorum* is under the control of multiple signaling pathways; our study indicates the sclerotial development also requires many structural proteins. Furthermore, as the first described fungal protein to contain PAN modules, Ss-Sl2 may provide important clues to determine the functions of similar proteins in other fungi.

## Materials and Methods

### Fungal strains and culture conditions

Wild type *S. sclerotiorum* isolate “SUN-F-M” used in this study was obtained from sunflower (*Helianthus annuus*) in Hohhot, Inner Mongolia, China and stored as mycelia plugs on PDA (Difco Laboratories, MI, USA) at 4°C. Strains were routinely subcultured on PDA to maintain vigor and purity. Transformants were cultured on PDA amended with 50 μg/ml hygromycin B (Calbiochem, San Diego, CA). To collect the hyphae from cultures grown on solid medium, a sterile cellophane membrane was placed on the medium before inoculation.

### Nucleic acid isolation

Mycelia were collected and frozen in liquid nitrogen and stored at −80°C. Genomic DNA was isolated according to a previously described method of Yelton et al. [47]. Total RNA was extracted with the Trizol reagent (Huashun Bioengineering Co, Shanghai, China) according to the manufacturer instructions.

### cDNA production and real-time RT-PCR

To evaluate the expression levels of *Ss-Sl2* transcripts in the different transformants containing pSisl2, the transformants and the wild type strain were cultured on PDA for 5 days. For the wild type strain, the sclerotia were in condensation stage after day 5. To evaluate the expression levels of *Ss-Hex1* and *Ss-Gpd* transcripts in the transformants containing pSihex1 or pSigad, the wild type and the transformants were cultured on PDA for 4 days. For the wild type strain, the sclerotia were in initial stage after day 4. The total RNA of these strains was extracted and treated with DNase 1 (RNase free) (Takara, Dalian, China). The treated RNA was used to synthesize the cDNA with the ReventAid^TM^ First Strand cDNA Synthesis Kit (MBI Fermentas, Flamborough, ON, Canada) according to the protocol specifications.

To evaluate the expression levels of *Ss-Sl2*, *Ss-Gpd*, and *Ss-Hex1*, relative quantification of gene expression was performed with the SYBR Green Realtime RT-PCR on a CFX96^TM^ Realtime System (Bio-Rad, Hercules, CA, USA). These pairs of primer were used: *Ss-Sl2* (RT-Ss-Sl2F 5′-TTCCCTCAGACTCAGCCTTAT-3′, RT-Ss-Sl2R 5′- GTAAATACTCCATTCCGTCCAT-3′), *Ss-Gpd* (RT-Ss-GpdF 5′-AATATGACTCCACTCACGGTCAAT-3′, RT-Ss-GpdR 5′- CCACCCTTCAAATGTGCCTTA-3), *Ss-Hex1* ( RT-Ss-Hex1F 5′-ATGAAGAAGCCGGAGCCC-3′, RT-Ss-Hex1R 5′ -GGCTTGGGTTGGCGATG-3′), and *actin* house-keeping gene (RT-actinF 5′ -GAGCTGTTTTCCCTTCCATTGTC-3′, RT-actinR 5′ -GACGACACCGTGCTCGATTGG-3′).

Dihydroxynaphthalene (DHN) is the precursor of melanin produced by *S. sclerotiorum* [48]–[50]. The DHN biosynthesis pathway is very common among many fungal species, and polyketide synthase (PKS) catalyses the first step of this pathway [51]. To understand if Ss-Sl2 is involved in the biosynthesis of melanin in *S. sclerotiorum*, a melanin biosynthesis associated polyketide synthase-encoding gene in *S. sclerotiorum* (*Ss-Pks1,* SS1G_13322) was identified. *Ss-Pks1*, the homology of *pksP* in *Aspergillus fumigates* [52], was obtained with a BLASTP analysis of genomic database of *S. sclerotiorum*. To study the expression levels of *Ss-Pks1* in the wild type strain and *Ss-Sl2* silenced strains, the following primers were designed: RT-Ss-Pks1F 5′ -ACTGCTACGCCGAAACCATC-3′, RT-Ss-Pks1R 5′ -CGCATAGGACCTGCCAACTC-3′.

These pairs of primer were designed with Primer Premier 5 software (Premier Biosoft International, Palo Alto, CA, USA) to obtain an amplification product of approximately 200 bp for each target gene. Real-time RT-PCR mixtures were composed of 10 μl of SYBR Green Realtime PCR Master Mix (Toyobo, Tokyo, Japan), 1 μl of cDNA, 4-pmol concentration of each primer, and nuclease-free water to a final volume of 20 μl. Total cDNA abundance in the samples was normalized using the actin gene as an internal control. Amplification conditions were as follows: 2 min at 95°C, then 40 cycles consisting of 20 s at 95°C, 15 s at 56°C, and 20 s at 72°C.

### Transmission electron microscopy and immunoelectron microscopy

Small pieces of tissue excised from different stages of *S. sclerotiorum* were fixed in a solution of 4% (vol/vol) glutaraldehyde in 100 mM phosphate buffer (pH 7.2) for 6 h at 4°C. Dehydration was preformed in a graded acetone series (from 30% to 100%). Samples were then embedded in Epon-821 and polymerized at 60°C as described previously [53]. Thin sections (50 nm) were cut on a Leica Ultracut UCT ultramicrotome with a diamond knife.

For immunogold labeling, ultrathin tissue sections were adhered to 400-mesh copper grids and floated on a drop of solution containing 1% (wt/vol) bovine serum albumin (Sigma-Aldrich, St. Louis, Mo.) in Tris-buffered saline (TBS) for 1 h. Anti-Ss-Sl2 antibodies were obtained with the prokaryotic expression system. Sections were then incubated for 2 h at room temperature with anti-Ss-Sl2 antibodies diluted 1/400 in TBS. After rinsing in TBS five times (10 min each time), the sections were incubated for 2 h with goat anti-rabbit immunoglobulin adsorbed to 10-nm gold particles (Biocell Research Laboratories, Cardiff, UK) and diluted 1/40 in TBS. The sections were rinsed in TBS five times and then in double-distilled water twice. After drying, the sections were incubated in uranyl acetate and observed with a JEOL JEM-1230 microscope at 80 kV. The specificity of immunogold labeling was detected by displacing anti-Ss-Sl2 antibodies with rabbit preimmune serum.

### Protein extraction and western blot analysis

The cytoplasm protein and cell wall protein of *S. sclerotiorum* were extracted as described by Pitarch et al. [54]. Protein concentrations were quantified with BCA Protein Assay Kit (Beyotime Biotech, Haimen, China). Western blot analysis was performed as described previously with minor modification [8], the total soluble protein of each sample loaded were 50 μg.

### Production of *Ss-Sl2* RNAi construct

To obtain the Ss-Sl2 RNAi vector, a vector named pCIT was constructed. In pCIT, a 350 bp intron from gene EAA75655.1 in *Gibberella zeae* was placed between the *N. crassa trpC* gene promoter (P*trpC*) and terminator (T*trpC*). Enzyme restriction sites *Pst*I and *BamH*I were added between the intron and P*trpC*, and *Hind*III and *Cla*I were placed between the intron and T*trpC* via some intermediate vectors. The primers Sisl2F (5′-CCC AAGCTT CTGCAG CAGCTTTCGAAGC-3′) and Sisl2R (5′-CC ATCGAT GGATCC AATAAGCCACCGAA-3′) were designed corresponding to nucleotides 311 to 620 of *Ss-Sl2* cDNA to amplify 310 bp of coding sequence. The restriction sites are underlined in the primer sequences, and were used to facilitate cloning. The 310-bp fragment was digested by the *Pst*I and *BamH*I and the excised fragment was ligated into pCIT, which was digested by the same enzymes to produce pCIT1. Then the *Ss-Sl2* gene fragment was digested by the *Hind*III and *Cla*I, and the excised fragment was ligated into pCIT1, which was digested by the same enzymes to produce pCIT2. The pCIT2 was then digested by *Sac*I and *Xho*I to obtain a fragment in which the two 310-bp fragments were inserted in opposite orientations downstream of the *N. crassa* P*trpC*. In the last step, the fragment digested by *Sac*I and *Xho*I was ligated into pCAMBIA3300, in which a bacterial hygromycin B phosphotransferase gene (*hph*) from pUCATPH [55] has been inserted at the enzyme recognition site *Xba*I. The resulting RNAi construct, pSisl2, was used to transform *S. sclerotiorum*.

### Production of *Ss-Gpd* and *Ss-Hex1* RNAi construct

*Ss-Gpd* RNAi vector was constructed according to the strategy of Nguyen [56]. Based on the *PtrpC*-*hph*-*TtrpC* fragment from pUCATPH, the *Aspergillus nidulans gpd* promoter (P*gpd*) from pAN7-2 [57] and the *ccdB* gene fragment with *Xcm*I cutting site from pGXT [58] were used to replace the *TrpC* and *hph* fragments, respectively, through some intermediate vectors. In this *PtrpC*-*ccdB*-*Pgpd* fragment, the *PtrpC* and *Pgpd* were placed in opposite directions. Then the *PtrpC*-*ccdB*-*Pgpd* fragment was inserted into pCXSN [58] to produce pCXDPH, an *hph* gene from pUCATPH was used to be the selection marker. To produce the *Ss-Gpd* RNAi vector, a 538-bp fragment of the *Ss-Gpd* gene was amplified with the primers SigapdhF (5′-CCAATGTACGTCATGGGTGTC-3′) and SigapdhR (5′- CGAAGTTCTTGTTGAGGGAGA-3′) and ligated into pCXDPH, which had been digested by *Xcm*I (New England Biolabs, Beverly, MA, USA) to produce pSigpd. In the same way, a 465-bp fragment of *Ss-Hex1* was amplified with the primers Sihex1F (5′- GTTACCATCCCTTGCCACCAC-3′) and Sihex1R (5′-GGCGATCTCACGACCTCCAT-3′), and ligated into pCXDPH with the same enzyme, *Xcm*I, to produce pSihex1.

### Transformation and evaluation of transformant strains

A method of *Agrobacterium*-mediated transformation was used to transform *S. sclerotiorum*. A fresh colony of *A. tumefaciens* EHA105 containing pSisl2, pSigpd or pSihex1 was cultured overnight at 28°C in LB liquid medium. Then the *A. tumefaciens* cells were diluted in minimal medium [59] amended with 50 μg/ml kanamycin and incubated overnight at 28°C. The *A. tumefaciens* cells were diluted to an optical density and cultured at 28°C in induction medium [59] for 6 h with gentle shaking. For co-cultivation, *A. tumefaciens* cells and fresh *S. sclerotiorum* mycelial plugs were cultured on a cellophane membrane placed on co-induction medium (induction medium with agar). After co-cultivation at 20°C for 2 days, the mycelial plugs on the cellophane membrane were removed, and the membrane with the fresh *S. sclerotiorum* mycelia and *A. tumefaciens* cells were transferred to a selective medium (PDA amended with 50 µg/ml hygromycin B and 200 µg/ml cefotaxime sodium (DingGuo, Beijing, China)) and incubated at 20°C for 4 days. Colonies that were regenerated through the selective medium were transferred to PDA amended with 50 μg/ml hygromycin B. Transformants were cultured at least three times on PDA containing 50 μg/ml hygromycin B by using hyphal tips.

### Co-immunoprecipitation and LC/MS/MS

To investigate proteins that interact with Ss-Sl2, the wild type strain was cultured on PDA for 4 days and all the culture included hyphae and immature sclerotia were collected as described before and lysed in cell lysis buffer (Beyotime Biotech, Haimen, China). The concentration of the protein in the supernatant was determined by Bradford assay with bovine serum albumin (BSA) as a standard. A total of 1 mg of protein extract was then incubated with 2 μg of the Ss-Sl2 antibodies and incubated at 4°C overnight with gentle shaking. Protein extract incubated with pre-immune serum was used as a control. Then 20 μl protein A + G agarose (Beyotime Biotech, Haimen, China) was added to the reaction mixtures and shaken at 4°C for 3 h. The agarose was then washed with PBS. The bound proteins were released in sodium dodecyl sulfate (SDS) gel sample buffer and analyzed by 10% SDS polyacrylamide gel electrophoresis. After separation on a polyacrylamide gel, the whole bands were digested with trypsin. The resultant peptides were analyzed using a capillary column liquid chromatography (LC)-microelectrospray mass spectrometry (MS) system with a QTRAP3200 mass spectrometer (Applied Biosystems, CA, USA) and a TEMPO nano LC Systems. Sequences obtained from MS/MS spectra were queried against the protein database created from the translation of the 14,522 predicted genes of *S. sclerotiorum* (www.broadinstitute.org/annotation/genome/sclerotinia_sclerotiorum/MultiDownloads.html).

### Yeast two-hybrid system

The yeast two-hybrid analysis was carried out using a GAL4-based yeast two-hybrid system-Matchmaker™ Gold Systems (Clontech, Palo Alto, CA). The *Ss-Sl2* region code for aa 17-352 (missing the 1–16 aa signal peptide) was amplified by adding two restriction sites, *Nde*I and *Sal*I. The resulting PCR fragment was cut with *Nde*I and *Sal*I and ligated into pGBKT7 to construct a bait. The full length cDNA of candidate proteins obtained by co-immunoprecipitation were cloned into pGADT7 to construct prey plasmids. The primers and restriction sites used to create these constructs are listed in Table 2. To test the specificity of the interaction, the bait plasmid and the prey plasmids were co-transformed into yeast strain Y2HGold (Clontech, Palo Alto, CA). The transformants were assayed for growth on SD (synthetic dropout)/-Trp-Leu-His-Ade plates and SD/-Trp-Leu-His-Ade plates with X-α-gal for β-galactosidase test.

### Detection of the maintenance ability of cellular integrity

According to previously described methods of Tenney et al. [27] and Soundararajan et al. [29], strains were cultured on medium with sorbose or under hyperosmotic stress for examination of the maintenance ability of cellular integrity of hyphae. The hyphal growth rates of strains on PDA and PDA with 2% to 5% sorbose, 5% NaCl, 1.2 M sucrose or 1 M sorbitol were measured respectively to determine the inhibition of hyphal growth. For observing the phenotype of hyphal tips and forming branches, the strains were cultured on PDA with 5% sorbitol for two days. Each experiment was repeated at least three times.
